# Evaluation of efficacy, safety and tolerability of Ambrisentan in Chinese adults with pulmonary arterial hypertension: a prospective open label cohort study

**DOI:** 10.1186/s12872-016-0361-9

**Published:** 2016-10-22

**Authors:** Y. Huo, Z. C. Jing, X. F. Zeng, J. M. Liu, Z. X. Yu, G. C. Zhang, Y. Li, Y. Wang, Q. S. Ji, P. Zhu, B. X. Wu, Y. Zheng, P. P. Wang, J. Li

**Affiliations:** 1 Cardiovascular, 1st Affiliated Hospital of Peking University, No. 8 Xishiku Street, Xicheng District Beijing, 100034 China; 2 State Key Laboratory of Cardiovascular Disease, FuWai Hospital, Chinese Academy of Medical Science & Peking Union Medical College, Beijing, China; 3 Rheumatology and Immunology, Peking Union Medical College Hospital, Beijing, China; 4 Pulmonary Circulation, Shanghai Pulmonary Hospital, Tongji Univeristy, Shanghai, China; 5 Cardiovascular, Xiangya Hospital Central-South University, Hunan, China; 6 Cardiovascular, Wuhan Asia Heart Hospital, Hubei, China; 7 Rheumatology, The 2nd Affiliated Hospital of Harbin Medical University, Harbin, China; 8 Cardiovascular, Beijing Shijitan Hospital, Beijing, China; 9 Ministry of Public Health & Department of Cardiology, Key Laboratory of Cardiovascular Remodeling and Function Research, Chinese Ministry of Education and Chinese Qilu Hospital, Shandong University, Shandong, China; 10 Department of Clinical Immunology, 1st Affiliated Hospital of the Forth Military Medical University, Shaanxi, China; 11 Cardiovascular, The 2nd Affiliated Hospital of Harbin Medical University, Harbin, China; 12 Cardiovascular, 1st Hospital of Jilin University, Changchun, China; 13 GlaxoSmithKline, Pudong Shanghai, China

**Keywords:** Ambrisentan, Chinese, Exercise capacity, Pulmonary arterial hypertension

## Abstract

**Background:**

Although several new drugs have been approved in recent years, pulmonary arterial hypertension (PAH) remains a rapidly progressive disease with a poor prognosis. Ambrisentan, a selective endothelin type A antagonist, has been approved for treatment of PAH. This open label study assessed the efficacy and safety of ambrisentan in Chinese subjects with PAH.

**Methods:**

Eligible patients with PAH (World Health Organisation [WHO] functional class [FC] II orIII) were enrolled and received Ambrisentan (5 mg) once daily for a 12-week preliminary evaluation period, and a 12-week dose-adjustment period (dose titration to 10 mgallowed). Endpoints included: change from baseline in 6-Minute Walk Distance (6-MWD), N-Terminal Pro B-Type Natriuretic Peptide (NT-pro-BNP), WHO FC, Borg Dyspnoea Index (BDI), clinical worsening of PAH and incidences of adverse events (AE).

**Results:**

One hundred thirty-three subjects (85 % women, mean age: 36 years) with PAH (WHOFC II or III) were enrolled and received ambrisentan (5 mg) once daily for a 12-week preliminary evaluation period, and a 12-week dose-adjustment period. Mean (SD) duration of drug exposure was 161.7 (27.13) days. Ambrisentan (average daily dose of 6.27 mg) significantly improved exercise capacity (6MWD) from baseline (mean: 377.1 m [m]) at week 12 (+53.6 m, *p* < 0.001) (primary endpoint). Improvement in exercise capacity was noted as early as week 4, and was sustained up to week 24 (+ 64.4 m, *p* < 0.001). NT-pro-BNP plasma levels decreased significantly (*p* < 0.001) at week 12 (−861.4 ng/L) and week 24 (−806 ng/L) from baseline (mean: 1600.7 ng/L). The WHO FC showed improvements for 44 subjects at week 12 and 51 subjects at week 24. BDI scores decreased significantly at week 12 (−0.3, *p* < 0.001) and week 24 (−0.2, *p* = 0.003) from baseline (mean: 2.5). Four patients died during the study (sudden cardiac death [*n* = 2], cerebral haemorrhage [*n* = 1], cardiac failure [*n* = 1]). Drug related adverse events occurred in 34.3 % of subjects; peripheral oedema (11.2 %) and flushing (8.2 %) occurred most frequently.

**Conclusion:**

Ambrisentan (5 and 10 mg, orally) significantly improved the exercise capacity in Chinese PAH subjects with a safety profile similar to that observed in global studies.

**Trial registration:**

NCT No. (ClinicalTrials.gov): NCT01808313; Registration date (first time): February 28, 2013.

**Electronic supplementary material:**

The online version of this article (doi:10.1186/s12872-016-0361-9) contains supplementary material, which is available to authorized users.

## Background

Pulmonary arterial hypertension (PAH) is a fatal vascular disease that entails a complex, multifactorial pathophysiology. It is characterised by vascular remodelling and a progressive increase in pulmonary vascular resistance, which eventually leads to right ventricular failure and premature death [1, 2]. An estimated incidence of 2 cases per million individuals every year has been reported for PAH [3]. Life expectancy of untreated PAH patients is not more than 2–5 years, with 1-year mortality rate of 15 to 32 % ([3–5]). The majority of the deaths reported in PAH patients are either due to sudden death or right-sided heart failure [6].

The therapeutic armamentarium for PAH is evolving continuously to explore new targets involved in pathobiological pathways of the disease process. At present, there are 4 categories of drugs approved globally for treating PAH: 1) prostaglandin analogues – act via prostacyclin pathway, 2) phosphodiesterase type-5 inhibitors - act via nitric oxide pathway, 3) endothelin receptor antagonist (ERA) – act via endothelin pathway [2] and 4) soluble cGMP stimulator–act via NO pathway. Ambrisentan, an endothelin –A (ETA) receptor- selective antagonist, is approved in the United States (US) (http://www.accessdata.fda.gov/), European Union (EU) (http://www.ema.europa.eu/) and many other countries to treat PAH patients with World Health Organisation (WHO) functional class (FC) II or III symptoms at oral doses of 5 and 10 mg once daily. Ambrisentan demonstrated significant efficacy on exercise capacity [7] with a survival rate of 94 % and a freedom from clinical worsening of 83 % at 1 year follow up [8]. The safety profile of ambrisentan is well-established and generally includes adverse events of peripheral oedema, nasal congestion, sinusitis, flushing, palpitations, nasopharyngitis, abdominal pain and constipation [9]. The adverse events associated with ambrisentan treatment are mostly of mild or moderate severity; Elevations of liver aminotransferases have been reported with ambrisentan and serious liver injury has been reported with related drugs, however in ARIES-1 and ARIES-2 studies, the reported incidence of aminotransferase elevations >3 × upper limit of normal (ULN) were 0 % on ambrisentan and 2.3 % on placebo. In ARIES-E study, twelve patients experienced ALT/AST >3 × ULN during the 2-year period [7, 8]. Ambrisentan may cause fetal harm if taken during pregnancy [9].

The pivotal studies that have been reported for ambrisentan were predominantly performed in white/caucasian population, the proportion of Asians being very low. Large variations in metabolism, efficacy and safety profile of drugs have been noted among individuals from varied racial and ethnic groups [10], suggesting that the clinical response to a drug therapy may change based on the ethnic/racial differences. Therefore, it becomes very important to account for these differences when clinically managing individuals from different ethnicities to provide a rationale therapeutic approach. Lack of big scale population based clinical data for ambrisentan in the Asian population has triggered more ethnic-specific clinical trials to identify specific therapeutic needs in this population.

This study investigated the efficacy and safety of ambrisentan in subjects with PAH from China where it has been conditionally approved for the treatment of WHO FC II or III PAH. The results of this study add to the existing clinical information for ambrisentan in Asians (specifically Chinese), which has been relatively scarce.

## Methods

### Study design

This open label, phase IIIb, single-arm study was conducted from 21 December 2012 to 15 August 2014 at 12 centres in China. Following a 4-week screening period, eligible subjects received 5 mg ambrisentan orally once daily for a 12-week primary evaluation period. Subjects then proceeded to a 12-week dose adjustment period during which dose titration to 10 mg was allowed. The total duration of study was 28 weeks.

### Study population

Chinese subjects of either gender, aged 18–75 years, diagnosed with PAH (WHO FC II or III) categorised in Group 1 of the WHO Updated Clinical Classification of Pulmonary Hypertension [1] were enrolled in the study. All subjects had to perform the 6 min walk test (6MWT) with a minimum distance of 150 m and maximum distance of 450 m. Additionally, results from a right heart catherisation performed within 6 months of screening had to demonstrate mean pulmonary artery pressure ≥25 mmHg; pulmonary vascular resistance ≥240 dyn/s/cm^5^; and pulmonary arterial wedge pressure or left ventricular end-diastolic pressure ≤15 mmHg. Additional requirements at entry into the study were total lung capacity ≥60 % and forced expiratory volume per second (FEV_1_) ≥55 % of predicted normal values.

The key exclusion criteria were: serum alanine aminotransferase (ALT) or aspartate aminotransferase (AST) values greater than 2 times ULN, serum bilirubin value greater than 1.5 times ULN, haemoglobin concentration <10 g/dL or haematocrit <30 %, severe hypotension (diastolic blood pressure <50 mmHg or systolic blood pressure <90 mmHg), clinically significant aortic or mitral valve disease, pericardial constriction, restrictive or congestive cardiomyopathy, life-threatening cardiac arrhythmias, left ventricular ejection fraction <45 %, left ventricular outflow obstruction, symptomatic coronary artery disease, autonomic hypotension or fluid depletion, and severe hepatic and renal impairment.

Also subjects who received prohibited medications including marketed ambrisentan, bosentan, phosphodiesterase −5 inhibitor, prostanoids, intravenous inotropes, and nitric oxide were excluded from the study. The use of calcium channel blockers (CCB) and statins were permitted only if the subjects were on stable doses for at least 4 and 12 weeks, respectively, before screening. Women of childbearing potential had to use medically acceptable method of contraception (such as hormonal method, intrauterine device, barrier methods such as condom or occlusive cap) during the study; pregnant and lactating women were excluded.

The study was conducted in accordance with ICH GCP and all applicable patient privacy requirements, and, the ethical principles that are outlined in the Declaration of Helsinki. The study has received ethical approval from multiple Independent Ethnics Committees (IECs) and that the name of these can be found in Additional file 1. The protocol and protocol amendments were also approved by institutional ethics committee of each study centre. Written informed consent was obtained from subjects prior to start of the study, the names of all IECs can be found in the Additional file 1.

### Assessments

The primary efficacy endpoint was the change from baseline to week 12 in the exercise capacity, as measured by the 6 min walk distance (6MWD) [11–13]. Additionally, the change from baseline to week 24 in 6MWD and the changes from baseline to week 12 and 24 in NT-proBNP plasma levels, WHO FC, and BDI score were assessed as secondary endpoints [14–16]. Echocardiography assessments were also performed, which included evaluation of prognostic factors such as pericardial effusion, tricuspid annular plane systolic excursion (TAPSE) and eccentricity index. Efficacy parameters (6MWD, WHO FC and BDI) were evaluated at the following time points: day 0 (predose [baseline]) and week 4, 8, 12, 16, 20 and 24 postdose. NT-pro BNP plasma levels and echocardiography parameters were assessed at baseline, week 12 and 24. Safety assessments included evaluation of time to clinical worsening of PAH, which was defined as the time from baseline to the first occurrence of death, lung transplantation, hospitalisation for PAH treatment, atrial septostomy or ambrisentan discontinuation due to change to other PAH treatment. Additionally, incidences and severity of adverse events (AEs), laboratory evaluations, liver function test, vital sign measurements, 12-lead electrocardiogram and physical examination were performed.

### Statistical analysis

Assuming a dropout rate of 10 % and a study power of 80 %, a proposed sample size of 104 evaluable subjects was considered sufficient to detect a clinically and statistically significant change in 6MWD from baseline to week 12 of 23 m with a standard deviation of 83 m [7]. Improvement in 6MWD was assessed using paired *T*-test, plasma NT-proBNP levels and BDI were assessed using Wilcoxon Signed-Rank test. WHO FC and echocardiography outcomes were summarised descriptively. Last observation carry forward (LOCF) method of imputation for missing data was used for 6MWD, WHO FC and BDI evaluations. The clinical worsening events were summarised and the Kaplan-Meier analysis was performed. All the safety data was summarised descriptively. Subgroup analyses for the efficacy data was performed based on gender, subjects having PAH associated with connective tissue diseases, and subjects receiving 10 mg ambrisentan treatment.

The Intent-to-treat (ITT) population was used for analysis of efficacy and demographic/baseline characteristics. It included all subjects who received at least one dose of ambrisentan and had an efficacy assessment performed both at baseline and after administration of the ambrisentan. The safety population comprised of all subjects who received at least one dose of ambrisentan.

An adhoc analysis was performed to determine the effect of ambrisentan on heart rate recovery at 1 min (HRR_1min_), 2 min (HRR_2 min),_ and 3 min (HRR_3min_) after exercise. HRR_1min,/2 min/3 min_ is defined as the difference in heart rate at the end of 6MWT and at 1/2/3 min after completion of the 6MWT.

## Results

### Patient disposition and baseline characteristics

Of the 134 subjects, 133 were included in the ITT population set (one subject was not included in ITT population due to no efficacy assessment performed after treatment). A total 10 out of 133 subjects (7.5 %) withdrew from the study. The majority of the withdrawals were due to AEs (*n* = 5) which are detailed in the safety section. The study population mainly comprised of women (85 %), with mean age of 36 (SD 10.25) years. PAH associated with connective tissue disease was the most prevalent diagnosis at baseline; subjects either had WHO FC II or III symptoms (Table 1). Concomitant medications were used pre-treatment and on-treatment by 76.9 and 85.1 % of subjects respectively, the most frequently used medications pre-treatment and on-treatment were same (furosemide, spironolactone, digoxin). Only one (0.7 %) subject was taking CCB during the study period.

**Table 1:** Demographic and baseline characteristics (ITT population)

	Ambrisentan (*N* = 133)
Age, years, mean (SD)	36 (10.3)
Gender, *n* (%)	
Men/women	20 (15)/113 (85)
Ethnicity, *n* (%)	
Not Hispanic or Latino ethnicity	133 (100)
BMI (kg/m^2^), mean (SD)	21.5 (3.2)
PAH classification, *n* (%)	
PAH associated with connective tissue disease	71 (53.4)
Idiopathic PAH	47 (35.3)
PAH associated with congenital heart disease	12 (9.0)
Heritable PAH	3 (2.3)
6MWD, m, mean (SD)	377.1 (61.3)
WHO PAH functional classification, *n* (%)	
Class II	70 (52.6)
Class III	63 (47.4)
BDI, mean (SD)	2.5 (1.4)
NT-ProBNP (ng/L), mean (SD)	1600.7 (1832.84)

*BMI* body mass index, *PAH* pulmonary arterial hypertension, *6MWD* 6-Minutes Walk Distance, *WHO* World Health organization, *NT-proBNP* N-terminal pro B-type natriuretic peptide, *BDI* Borg Dyspnoea Index, *ITT* intent-to-treat

All subjects (*n* = 134) received at least a single dose of ambrisentan. Overall, mean (SD) duration of exposure was 161.7 (27.13) days, and the average daily dose was 6.27 (1.24) mg (Table 2).

**Table 2:** Extent of drug exposure during study

Exposure		Ambrisentan (*N* = 134)		
		Primary Evaluation Period	Dosage-adjustment Period	
			Combined Ambrisentan^a^	Ambrisentan 10 mg
Exposure (days)	*n*	134	127	73
	Mean (SD)	83.0 (9.13)	82.8 (5.59)	75.8 (16.02)
	Median	85.0	83.0	83.0
	Min, Max	31, 90	34, 94	27, 87
Range of exposure, *n* (%)	*n*	134	127	73
	<=28 days	0	0	2 (2.7)
	29–56 days	5 (3.7)	2 (1.6)	12 (16.4)
	57–84 days	37 (27.6)	90 (70.9)	44 (60.3)
	> = 85 days	92 (68.7)	35 (27.6)	15 (20.5)
Average of daily dose (mg)	*n*	134	127	73
	Mean (SD)	4.95 (0.170)	7.81 (2.462)	10.11 (0.346)
	Median	4.94	9.04	10.12
	Min, Max	4.4, 6.5	4.8, 11.5	8.5, 11.5

^a^Combined for all subjects who received Ambrisentan 5 mg and Ambrisentan 10 mg in Dosage adjustment Period

### Efficacy

**Fig. 1:**
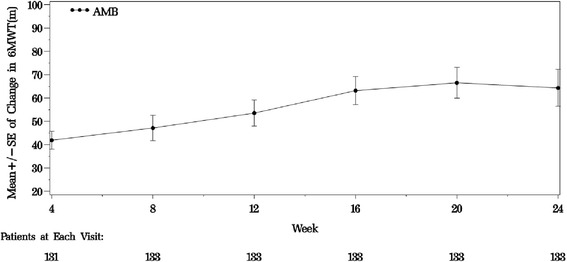
Improvement in 6MWD over 24 weeks following ambrisentan treatment (LOCF) (ITT population). Note: Mean (SD) baseline value for 6MWD was 377.1 (61.30) meters. *AMB*: ambrisentan

A significant increase in 6MWD from baseline (mean 377.1 m) was noted after 12 (53.6 m, *p* < 0.001) and 24 (64.4 m, *p* < 0.001) weeks of ambrisentan treatment (Table 3). A significant improvement in 6MWD was noted as early as week 4 and continued for all subsequent time points (Fig. 1 and Table 3). Plasma NT-proBNP levels decreased significantly with ambrisentan treatment at week 12 (−861 ng/L, *p* < 0.001) and week 24 (−806 ng/L, *p* < 0.001) compared to baseline (mean 1601 ng/L), both *p* <0.001.

**Table 3:** Change from baseline in 6MWD, BDI scores, WHO functional classification and NT-proBNP levels after ambrisentan treatment (ITT population)

Efficacy measures	Baseline	Week 12	Week 24
6MWD, meters (LOCF)			
*N*	133	133	133
Mean (SD)	377.1 (61.30)	+53.6 (64.50)	+64.4 (91.17)
*P value (95 % CI)		<0.001 (42.5; 64.7)	<0.001 (48.7; 80.0)
BDI scores (LOCF)			
*N*	133	133	133
Mean (SD)	2.5 (1.37)	−0.3 (1.52)	−0.2 (1.95)
*P* value (95 % CI)		<0.001	0.003
WHO functional classification, *n* (%) (LOCF)			
*N*	-	133	133
Improved by class 1/2		44 (33.1)/0	51 (38.3)/0
No change		84 (63.2)	77 (57.9)
Worsened by class 1/2		4 (3.0)/1 (0.8)	3 (2.3)/2 (1.5)
NT-proBNP levels, ng/L (Observed data)			
*N*	132	123	122
Mean (SD)	1600.7 (1832.84)	−861.4 (1452.96)	−806.0 (1384.00)
*P* values (95 % CI)		*P* < 0.001	*P* < 0.001

*LOCF* last observation carry forward, *6MWD* 6-Minutes Walk Distance, *BDI* Borg Dyspnoea Index, *WHO* World Health Organisation, *ITT* intent-to-treat

*P-value calculated from paired *T*-test. *P*-value was derived from Wilcoxon signed-rank test. *P* < 0.05 indicated statistically significant difference from baseline

A large proportion of subjects showed improvement in the WHO FC from baseline; 44 subjects (33.1 %) at week 12 and 51 subjects (38.3 %) at week 24 showed an improvement by 1 class. Only 5 subjects showed worsening of functional class by 24 weeks of treatment. Significant improvement in BDI scores was observed at week 12 (decrease of 0.3 score, *p* < 0.001) and at week 24 (decrease of 0.2 score, *p* = 0.003) (Table 3). Echocardiography parameters showed a trend towards improvement at week 12 and 24 with ambrisentan treatment. A decrease (improvement) in pericardial effusion volume from baseline was observed for 13 (12.0 %) subjects at week 12 and for 18 (16.7 %) subjects at week 24. About 65 % of subjects showed no change in effusion volume at week 12 and 24; few subjects (5 to 9 %) showed worsening in pericardial effusion. Mean change (SD) in tricuspid annular plane systolic excursion was +0.14 (0.31) at week 12 and +0.15 (0.32) at week 24 compared to baseline (mean 1.55 (0.33)). Mean change (SD) in systolic eccentricity index was −0.07 (0.41) at week 12 and −0.13 (0.37) at week 24 compared to baseline (mean 1.90 (0.48)). Mean change (SD) in diastolic eccentricity index was −0.08 (0.24) at week 12 and −0.07 (0.22) at week 24 compared to baseline (mean 1.44 (0.25)).

Subgroup analyses showed that the overall efficacy pattern of ambrisentan in the subjects having PAH associated with connective tissue disease was similar to the pattern noted in overall population. The primary outcome measure of 6MWD was significantly (*p* < 0.001) increased by 63.8 m and by 73.2 m at week 12 and 24, respectively, following ambrisentan treatment in subjects with PAH associated with connective tissue disease. This increase was slightly greater than that noted for overall population. The subgroup of subjects receiving 10 mg dose of ambrisentan during dose-adjustment period showed significant improvement in 6MWD at week 12 (53.9 m [95 % CI: 41.7 to 66.1; *p* < 0.001]) and week 24 (69.7 m [95 % CI: 48.1 to 91.3; *p* < 0.001]) after treatment. The increase in 6MWD was similar to that noted for overall population. Subgroup analysis by gender showed that dmbrisentan achieved significant improvement in 6MWD, NT-ProBNP, WHO FC and BDIin both men and women. In general, men demonstrated a larger improvement from baseline in 6MWD compared with women. The 6MWD was significantly (*p* < 0.001) increased by 78.2 and 94.2 m in men and by 49.2 and 59.1 m in women at week 12 and 24, respectively. Improvements noted in other parameters were however larger in women than in men.

The HRR_1min, 2 min, 3 min_ after exercise was faster following ambrisentan treatment at week 12 and 24 than that noted at baseline (difference in heart rate over 1 to 3 mins post exercise ranged from 9.0 to 18.2 beats/min at baseline, 10.8 to 20.2 beats/min at week 12 and 11.7 to 21.2 beats/min at week 24). A significant decrease of the heart rate difference from baseline was noted only at 1 and 2 min post exercise after 24 weeks of ambrisentan treatment (change in heart rate from baseline: −2.7 and −3.3 beats/min at 1 and 2 mins post exercise at week 24, respectively; *p* < 0.05).

### Safety

**Fig. 2:**
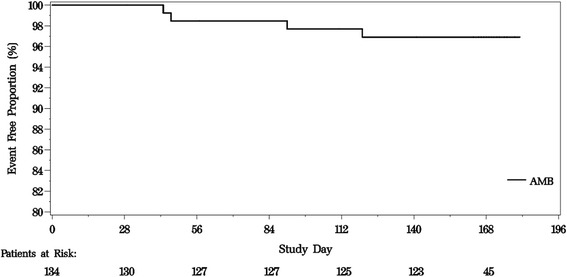
Kaplan-Meier plot for time to clinical worsening (safety population)

A total of 91 out of 134 subjects (67.9 %) experienced at least one AE during the study. As shown in Table 4, the most common AEs regardless of relationship to study drug were peripheral oedema, flushing, elevations of hepatic enzymes (ALT, AST, GGT) or bilirubin, headache, and nausea. Most of these events were of mild to moderate intensity. Drug-related events were noted in 46 out of 134 subjects (34.3 %), the most frequent were peripheral oedema (11.2 % [15 out of 134 subjects]) and flushing (8.2 % [11 out of 134 subjects]). The other individual drug-related events occurred in less than 3 patients each. Up to week 24, clinical worsening of PAH occurred in 4 subjects. More than 95 % of study population did not have worsening of PAH after 12 and 24 weeks of treatment (Fig. 2). Four deaths (including 1 subject died after 2 months of hospitalization for cardiac failure) were reported during the study, all were related to clinical worsening (sudden cardiac death [*n* = 2], cerebral haemorrhage [*n* = 1], cardiac failure [*n* = 1]). Five subjects discontinued the study due to AEs which included sudden cardiac death (*n* = 2), cardiac failure (*n* = 1), cerebral haemorrhage (*n* = 1), and generalized oedema (*n* = 1); none of these events were considered related to ambrisentan treatment. Eleven subjects (8.2 %) had a serious adverse event. Of these, only 1 event of peripheral oedema was considered related to ambrisentan treatment. No subjects experienced increases in ALT and AST levels >3 × ULN or bilirubin > 2 × ULN. No significant changes were noted in the hematology parameters. There were no clinically meaningful changes in vital signs, ECG and physical examination.

**Table 4:** Most frequent (≥5 %) adverse events during ambrisentan treatment (safety population) by maximum Intensity

Adverse events	Mild	Moderate	Severe	Total
Any adverse events, n (%)	52 (38.8)	32 (23.9)	7 (5.2)	91 (67.9)
Oedema peripheral	10 (7.5)	5 (3.7)	0	15 (11.2)
Flushing	10 (7.5)	1 (0.7)	0	11 (8.2)
Alanine aminotransferase increased	8 (6.0)	1 (0.7)	0	9 (6.7)
Aspartate aminotransferase increased	7 (5.2)	2 (1.5)	0	9 (6.7)
Blood bilirubin increased	8 (6.0)	1 (0.7)	0	9 (6.7)
Gamma-glutamyltransferase increased	4 (3.0)	3 (2.2)	0	7 (5.2)
Headache	7 (5.2)	0	0	7 (5.2)
Nausea	4 (3.0)	3 (2.2)	0	7 (5.2)

All the events of peripheral oedema and flushing were reported to be related to the study drug

## Discussion

This study conducted in Chinese subjects predominantly included subjects with PAH associated with connective tissue disease and WHO FC II or III symptoms. The majority of the population in this study was women and relatively young which was consistent with the earlier global studies for ambrisentan. Ambrisentan treatment in Chinese patients resulted in significant improvement in several important clinical parameters such as exercise capacity. In addition, over 24 weeks of ambrisentan treatment, more than 95 % of patients did not have clinical worsening of disease.

The 6MWD which measures the functional exercise capacity was employed as the primary endpoint in this study. Treatment with ambrisentan (5 mg) resulted in significant improvement from baseline in 6MWD at week12 (+53.6 [64.50] meters), incremental improvements were noted up to week 24 (+64.4 [91.17] meters). Dose adjustment was allowed upto 10 mg. Ambrisentan further showed improvement in Borg Dyspnoea Index, which is a visual analogue score used to quantify efforts during 6MWT. Given the progressive nature of the disease, the impact of ambrisentan on WHO FC was noteworthy even though inclusion criteria required a minimum of 150 m on the 6MWD and no greater than 450 m. The majority of subjects maintained or had an improvement in functional class after 12 and 24 weeks treatment; only 5 subjects experienced worsening of functional class by end of treatment period. The results corroborated the clinical worsening outcomes which showed about 95 % of subjects free from worsening after 12 and 24 weeks of treatment. The clinical benefits of ambrisentan on the aforementioned parameters (6MWD, WHO FC, BDI) were noted as early as week 4 and were sustained up to week 24.

Improvements in cardiac function were also evident. NT-proBNP plasma concentrations decreased significantly with ambrisentan treatment. Increased plasma NT-ProBNP levels are associated with right ventricular systolic dysfunction, and increased risk of early death [15]. There was a trend towards improvement in echocardiography parameters which may suggest a potential beneficial impact of ambrisentan treatment on heart function. The heart rate recovery at 1 and 2 min post exercise was faster following ambrisentan treatment at week 24. This observation was of particular interest, as heart rate recovery is known to be a strong predictor of clinical worsening and survival in subjects with PAH [17].

Overall the efficacy noted in this study was consistent with earlier pivotal studies in the western population (6MWD improved by 51 m with ambrisentan 10 mg) [7, 8, 18] and in studies conducted in Japanese populations (6 MWD improved by 33.5 with ambrisentan 10 mg) [19, 20]. Results of subgroup analyses conducted in this study with subjects diagnosed with PAH associated with connective tissue disease showed improvements in efficacy parameters (6MWD, WHO FC, BDI and NT-proBNP plasma levels) with ambrisentan treatment.

In terms of safety, most of the AEs observed in this study were either mild or moderate in intensity. The types and frequencies of AE for drug-related events were similar to those noted in earlier studies in western as well as Japanese populations [7, 8, 18–20]. Peripheral oedema was the most frequently reported drug- related event and was noted in 11.2 % of subjects. AEs leading to discontinuation were few and were generally related to disease progression. Four subjects experienced disease progression, which eventually led to death. Of note, none of the subjects had any liver function abnormalities of clinical concern. ALT and AST levels were below 3 fold the ULN even at the highest dose tested (10 mg). Based on the safety profile observed during the study, it appears that dose escalation to 10 mg could be successfully achieved in this Chinese population without any additional safety concerns. To a large extent, the safety outcomes including the decrease in clinical worsening with ambrisentan treatment in this Chinese population was similar to the studies conducted in western and Japanese subjects [7, 8, 18–20].

Although encouraging results have been obtained from this study, the lack of a comparator group poses some limitations on the conclusions that can be achieved. Using a placebo-controlled design would not have been ethically appropriate since there are approved PAH medications available in China, while using the only available active-control (bosentan) at the time of study start using a non-inferiority design was unot feasible due to the large sample size and differences in the approved indications for the two ERAs. Nevertheless, the efficacy and safety results obtained from this study were in line with the pivotal placebo-control trials in western populations.

## Conclusions

In this study, ambrisentan at doses of 5 and 10 mg showed significant improvement in the exercise capacity and clinical worsening did not occur in more than 95 % of patients after 24 weeks treatment in Chinese subjects. Ambrisentan demonstrated a safety profile similar to that observed in other populations, with no unexpected drug-related adverse events or any new safety signals noted during the study. Ambrisentan could be considered as an effective approach for management of PAH in Chinese subjects.

## Additional file

Additional file 1: Affiliations of all the ethics committees (IECs) that approved the study. (DOCX 13 kb)
