# Supplementary material for: Evaluation of efficacy, safety and tolerability of Ambrisentan in Chinese adults with pulmonary arterial hypertension: a prospective open label cohort study
Source: BMC Cardiovasc Disord. 2016 Oct 22;16:201. doi: 10.1186/s12872-016-0361-9 (PMC5075402; doi:10.1186/s12872-016-0361-9)
Supplement: Additional file 1: — Affiliations of all the ethics committees (IECs) that approved the study. (DOCX 13 kb) [file 12872_2016_361_MOESM1_ESM.docx]

**Affiliations of all the ethics committees that approved the study**

| **CHINA** | **IEC (independent ethics committees)** |
| --- | --- |
|  | Ethics Committee of Peking University First  Hospital |
|  | Ethics Committee of Fuwai Cardiovascular  Hopsital |
|  | Ethics Committee of Shandong University Qilu  Hospital |
|  | Medical Ethics Committee of the 2nd Affiliated  Hospital of Harbin Medical University |
|  | Ethics Committee of Shanghai Pulmonary  Hospital, |
|  | Medical Ethics Committee of Beijing Shijitan  Hospital |
|  | Hospital of Harbin Medical University |
|  | Ethics Committee of Xiangya Hospital of  Centre South University |
|  | Ethics Committee of Peking Union Medical  College Hospital |
|  | Ethics Committee of Wuhan Asia Heart  Hospital |
|  | Ethics Committee of the first Hospital of Jilin  University |
|  | Independent Ethics Committee, Affiliated  Hospital of Fourth Military Medical University |
